# Interatrial conduction delay in a young woman mimicking complete atrioventricular block

**DOI:** 10.1016/j.hrcr.2026.04.010

**Published:** 2026-04-15

**Authors:** Vito Mustapić, Martin Manninger, Daniel Scherr, Stefan Kurath-Koller, Kristijan Šipoš, Janko Szavits Nossan

**Affiliations:** 1 Magdalena Clinic for Cardiovascular Disease, School of Medicine, J.J. Strossmayer University in Osijek, Krapinske Toplice, Croatia; 2 Division of Cardiology, Department of Internal Medicine, Medical University of Graz, Graz, Austria; 3 Division of Pediatric Cardiology, Department of Pediatrics, Medical University of Graz, Graz, Austria; 4 Faculty of Dental Medicine and Health, J.J. Strossmayer University in Osijek, Osijek, Croatia; 5 Faculty of Medicine, J.J. Strossmayer University in Osijek, Osijek, Croatia

**Keywords:** AV block, Interatrial conduction delay, Interatrial dissociation, Bachmann bundle, Electrophysiological study, Cardiac surgery

**Key Teaching Points**

• Bachmann bundle is the dominant interatrial conduction pathway and is vulnerable to injury during atrial surgery or extensive ablation.
• Interatrial dissociation may present as apparent atrioventricular (AV) block, with dissociated P waves and narrow QRS complexes—true AV block should be differentiated with an electrophysiological (EP) study before pacemaker implantation.
• A comprehensive EP study demonstrating preserved AV nodal conduction is essential for correct diagnosis and management.

## Introduction

Interatrial conduction delay or dissociation, a rare phenomenon where the left atrium activates later than or independently of the right atrium, arises from disruption of interatrial connections. Bachmann bundle is the principal pathway between the atria and can be potentially damaged by surgical interventions in the right or left atrium or by extensive atrial ablations.^1^ We present a case of a young woman with a history of 2 mitral valve surgeries and right atrial ablation for postincisional atypical atrial flutter who presented with an apparent complete atrioventricular (AV) block on Holter monitoring that was ultimately attributed to interatrial conduction delay.

## Case report

A 31-year-old woman was referred for permanent pacemaker implantation after asymptomatic AV dissociation on a routine Holter electrocardiogram. Her medical history included bioprosthetic mitral valve replacement at 22 years of age for infective endocarditis after benign brain tumor surgery, followed by mechanical mitral valve replacement at 31 years of age owing to severe prosthetic stenosis.

2 months after her second valve surgery, she developed atypical atrial flutter, which was mapped using a CARTO™ 3-dimensional electroanatomic mapping system (Biosense Webster, Johnson & Johnson MedTech) to a posterolateral right atrial scar and the cavotricuspid isthmus (CTI). Ablation of the CTI and an intercaval line terminated the flutter, which was subsequently noninducible.

**Figure 1:**
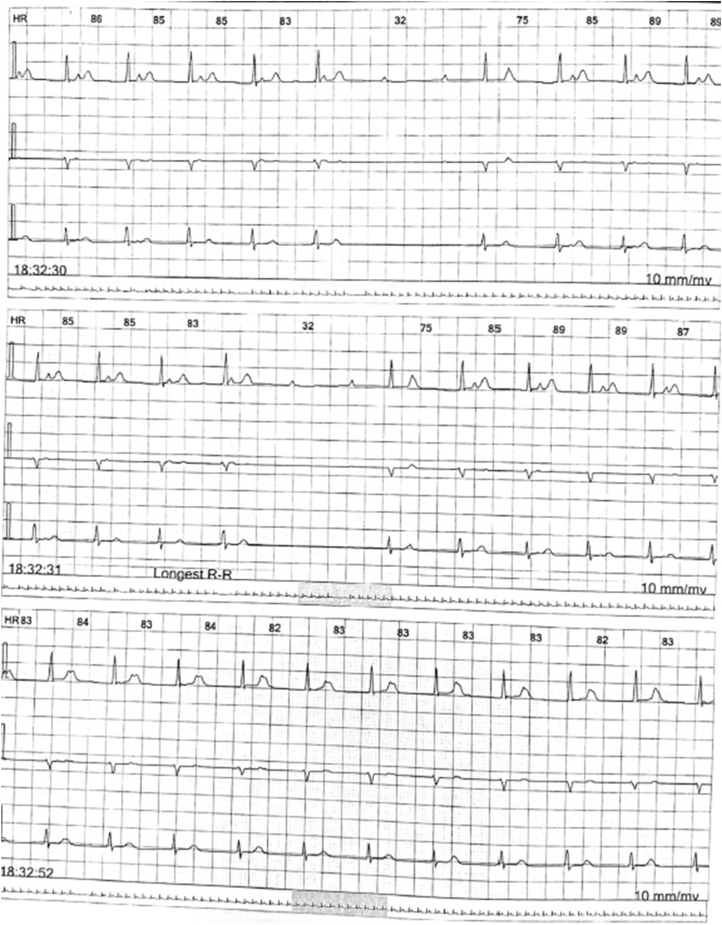
Holter electrocardiogram illustrating atrioventricular dissociation with narrow QRS complexes and stable ventricular response.

**Figure 2:**
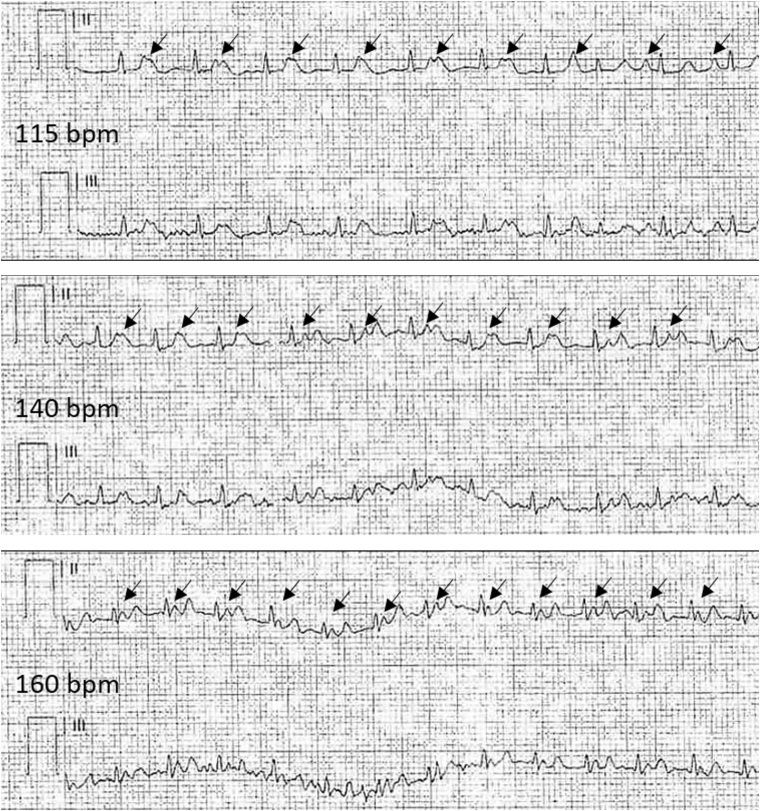
Treadmill test with increasing heart rate from 115 to 160 bpm and persistent atrioventricular dissociation (*arrow* pointing P waves). bpm = beats per minute.

4 months later, Holter monitoring demonstrated sustained AV dissociation for nearly 24 hours. The tracing showed narrow QRS complexes, ventricular rate ranging from 41 to 122 beats per minute (bpm), and no significant pauses (Figure 1). During an exercise test, her heart rate increased to 160 bpm with persistent AV dissociation (Figure 2). Considering the unexpectedly stable escape rhythm at this rate, along with the absence of significant pauses, further electrophysiological (EP) evaluation was undertaken before pacemaker implantation.

**Figure 3:**
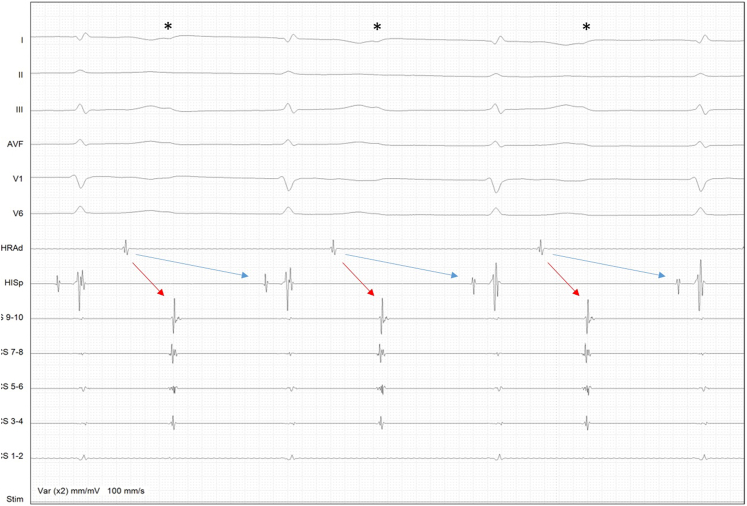
Intracardiac tracings from an electrophysiological study showing surface P waves matching CS activation (*asterisk* marking on surface electrocardiogram) and prolonged intraatrial (*blue arrow*) and interatrial (*red arrow*) conduction times. HRA signal presents activation of RA near sinus node and CS activation presents activation of the LA. Reference interval: AH 55–125 ms and HV 35–55 ms. CS = coronary sinus; HRA = high right atrium; LA = left atrium; RA = right atrium.

**Figure 4:**
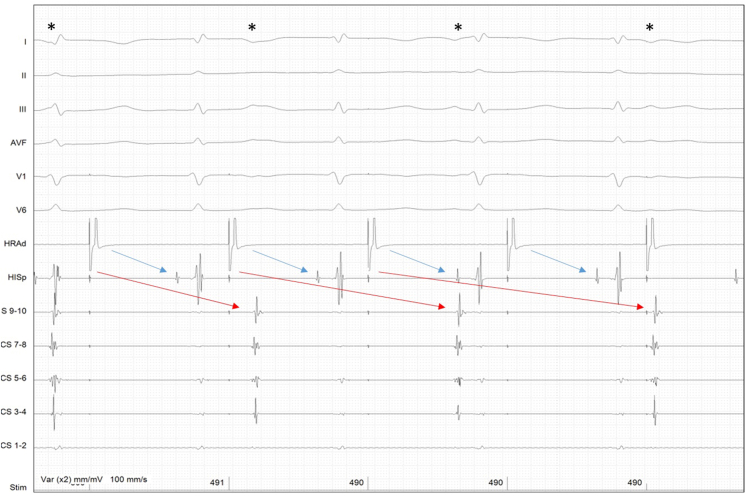
Incremental atrial pacing with 490 ms cycle length exhibits shortening of intraatrial conduction (*blue arrow*), preserved 1:1 sinoatrial node to atrioventricular node conduction (as seen with *blue arrow* showing every HRA signal is conducted to HIS and surface QRS) and further left atrial activation delay (*red arrow*) - surface P waves dissociated from QRS complexes at high rates (*asterisk* marking on surface electrocardiogram). Reference interval: AH 55–125 ms and HV 35–55 ms. HIS = His bundle region; HRA = high right atrium.

**Figure 5:**
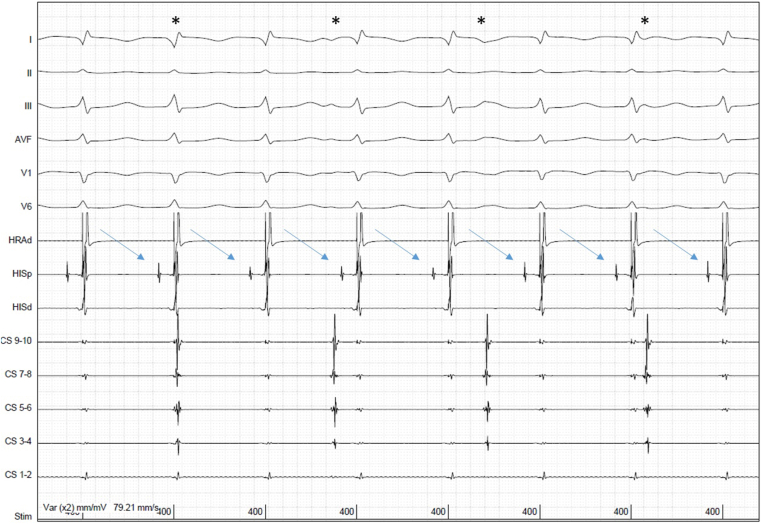
Incremental atrial pacing from HRA with 400 ms cycle length (150 beats per minute), 1:1 atrioventricular conduction as seen with *blue arrow* showing every HRA signal is conducted to HIS and surface electrocardiogram (*blue arrow*) and complete dissociation of the right and left atrium (appreciate the CS signals and P wave marked with *asterisk* on surface electrocardiogram completely dissociated from HRA signal). Reference interval: AH 55–125 ms and HV 35–55 ms. CS = coronary sinus; HIS = His bundle region; HRA = high right atrium.

Ultrasound-guided right femoral venous access was achieved, and catheters were positioned in the high right atrium (HRA), His bundle region, coronary sinus (CS), and apical right ventricle. Baseline intervals revealed prolonged intraatrial conduction (HRA to His bundle region 460 ms) and interatrial conduction (HRA to proximal CS 220 ms), with a normal HV interval of 45 ms and preserved 1:1 AV conduction (reference interval AH 55–125 ms and HV 35–55 ms). Surface P waves correlated with CS activation (Figure 3). During incremental atrial pacing, 1:1 AV nodal conduction persisted with Wenckebach at 150 bpm. At higher pacing rates, activation of the left atrium via delayed CS conduction went from Wenckebach (Figure 4) to complete interatrial dissociation (Figure 5), causing surface P waves to appear dissociated from the QRS complexes and simulating complete AV block. Given preserved AV nodal conduction and the absence of symptoms, pacemaker implantation was deferred. The patient remained asymptomatic at 1-year follow-up, with telemonitoring showing continued P–QRS dissociation and narrow QRS complexes.

## Discussion

Interatrial conduction depends predominantly on Bachmann bundle, although subsidiary pathways through the CS and fossa ovalis also contribute.^2^ Surgical or ablative damage to these pathways may cause interatrial dissociation, where right and left atria depolarize independently.^1^ This rare phenomenon has been predominantly reported after extensive atrial surgery, including the maze procedure or mitral valve interventions.^3^ There have been reports of interatrial dissociation after catheter ablation, albeit very extensive ablations in older and more diseased patients.^4^^,^^5^

**Figure 6:**
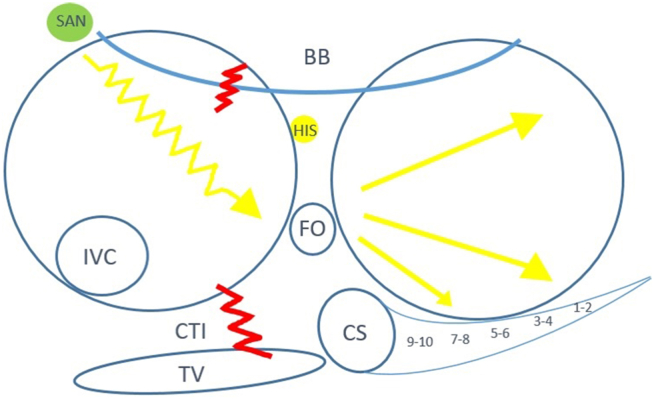
A presumed conduction scheme from the SAN to the left atrium (*yellow line*) with conduction block at the level of BB and CTI (*red line*). BB = Bachmann bundle; CS = coronary sinus; CTI = cavotricuspid isthmus; FO = fossa ovalis; HIS = His bundle region; IVC = inferior vena cava; SAN = sinoatrial node; TV = tricuspid valve.

In the present case, we propose that probable surgical damage from the second procedure disrupted Bachmann bundle. Subsequent CTI ablation likely disrupted the CS conduction route, leaving only the peri–fossa ovalis route patent, causing the left atrial activation delay, especially at higher rates (Figure 6). The left atrium, activated via delayed conduction from the right atrium, produced surface P waves decoupled from ventricular activation, giving the illusion of complete AV block.

Recognition of this finding is clinically important, given that misinterpretation may lead to unnecessary pacemaker implantation. EP evaluation is crucial when apparent AV block coexists with narrow QRS complexes and high “escape” rhythm, especially in patients with previous cardiac surgery or atrial ablation. Our patient’s preserved AV conduction and absence of symptoms highlighted that no pacing was required. If the patient develops symptoms, conduction system pacing—specifically a combination of Bachmann bundle pacing and left bundle branch area pacing—could provide an effective physiological alternative for the management of this rare conduction disturbance.

## Conclusion

This case illustrates that interatrial conduction delay can mimic complete AV block in patients with previous atrial surgery or extensive ablation. An EP study is essential to correctly identify this condition, ensuring appropriate management and avoiding unnecessary device implantation.

## Disclosures

The authors have no conflicts of interest to disclose.
